# Precipitation contributes to plant height, but not reproductive effort, for western prairie fringed orchid (*Platanthera praeclara* Sheviak & Bowles): Evidence from herbarium records

**DOI:** 10.1002/ece3.6647

**Published:** 2020-08-09

**Authors:** Lori A. Biederman, Sydney M. Weldon, Derek S. Anderson, Mark J. Leoschke

**Affiliations:** ^1^ Department of Ecology, Evolution and Organismal Biology Iowa State University Ames Iowa USA; ^2^ Minnesota Department of Natural Resources Saint Paul Minnesota USA; ^3^ Iowa Department of Natural Resources Des Moines Iowa USA

**Keywords:** Great Plains North America, museum collections, plant reproduction, Tallgrass prairie

## Abstract

The western fringed prairie orchid (WFPO) is a rare plant found in mesic to wet tallgrass prairies in the Great Plains and Midwest regions of the United States. The size of WFPO populations varies considerably from year to year, and studies have suggested that population size is dependent on precipitation during critical periods in the plant's annual development. We hypothesized that plant height and reproductive effort would also be controlled by precipitation, either during these periods or over a broader period. We acquired available images of WFPO from 21 herbaria, and of these 141 individual plants had information adequate for analysis, although some population/year combinations were represented multiple times. For each specimen, we measured plant height (cm) and reproductive effort (as measured by total flower and bud count). We used bootstrapped linear regression, randomly selecting one individual from each population/year combination, to compare precipitation models, both during critical periods and the various summaries. We found that precipitation during the phenologically critical periods was a poor predictor of plant height and reproductive effort. Of the broader precipitation variables, accumulated precipitation from January 1 to collection date best described plant height. We also used correlations to detect a relationship among the variables WFPO height, reproductive effort, precipitation, latitude, and year of collection. Year of specimen collection was negatively correlated with WFPO plant height and accumulated precipitation, suggesting that both have declined in more recent years. Negative correlations with latitude also suggest height and precipitation decrease in the northern part of WFPO's range. Reproductive effort was not related to tested precipitation variables; however, it was weakly correlated with plant height. Although the results are limited, this study leverages available data and makes inferences on WFPO biology over broad ranges of time (1894–2012) and latitude (37.5°–49.9°).

## Graphical abstract

Using herbarium specimens from a broad range of time (1894–2012) and latitude (37.5°–49.9°), we consider the influence of precipitation on the height and reproductive effort for *Platanthera praeclara* (western prairie fringed orchid). We found that precipitation during phenologically significant periods was a poor predictor of plant height, but accumulated precipitation from 1 January to collection date best described plant height. Reproductive effort, as measured by flower and bud number, was not affected by environmental conditions and only weakly correlated with plant height.

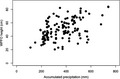

## 1 INTRODUCTION

*Platanthera praeclara* (western fringed prairie orchid (WFPO)) is a perennial orchid that is endemic to tallgrass mesic to wet prairies in the Great Plains and Midwest regions of the United States. Both charismatic and rare, this species has been the subject of much research on its life history (including Alexander, Kirby, Biondini, & Dekeyser, 2010; Borkowsky & Westwood, 2009; Cuthrell & Rider, 1993; Erickson, Lym, & Kirby, 2006; Fauske & Rider, 1996; Friesen & Westwood, 2013; Jordan, Fauske, Harris, & Lenz, 2006; Ross, Aldrich‐Wolfe, Lance, Glenn, & Travers, 2013; Sieg, King, Miller, & Nicholas, 1998; Travers, Fauske, Fox, Ross, & Harris, 2011). Despite this large body of work, many aspects of WFPO's life history remain elusive. For example, individual WFPO plant size and reproductive effort, as estimated by flower production, vary considerably within and among populations (Biederman, *personal observation*).

WFPO can grow up to 88 cm with a terminal raceme having up to 33 white flowers (Bowles, 1983; Smith, 2012). Historically, its range extended from southern Manitoba, Canada, to northeastern Oklahoma in the United States, but it is presumed extirpated in Oklahoma and South Dakota. This region experiences a continental climate, characterized by warm humid summers and cold–dry winters. Notably, there is great fluctuation in temperature throughout the year, with an annual temperature variation of 43°C in the southern portion of the species' range and 50.3°C in its northern part of its range (Young et al., 2011).

Plants, in general, grow taller and are more productive when current yearly conditions, such as soil moisture, nutrients, and sunlight, are ideal for growth. Perennial plants can also store resources and use them at a later time. WFPO, specifically, simultaneously invests resources into current production while also provisioning for the next growing season by developing a storage tuber from which next year's plant arises (Smith, 2012). Presumably larger tubers will support larger plants and those large plants will produce reproductive structures (Sather & Anderson, 2010).

Previous studies have found that number of flowering plants in a year relies on precipitation during critical phenological periods. For example, Willson, Page, and Akyuz (2006) analysis used data from a single WFPO population at Pipestone National Monument in Minnesota US (44° Latitude) over 8 years (1995–2004, excluding 2 years with prescribed burns) and the precipitation model that best explained total flowering plant population size was *y* = 196.73 + 7.28 previous senescence − 9.30 dormancy (adj. *R* ^2^ = .77). Furthermore, Morrison, Haack‐Gaynor, Young, and Debacker (2015) also used data from the single population at Pipestone National Monument in Minnesota, United States (44° Latitude), from 1995 to 2012, including 3 years with prescribed burns. Although several candidate models were similar, the precipitation model that best explained total flowering plant population size was *y* = −319.11 + 13.12 previous mature + 12.07 emergence (adj. *R* ^2^ = .68).

Bleho, Koper, Borkowsky, and Hamel (2015) also predicted flowering plant population size from climate. They used data from between to 61 and 277 metapopulations within ~65 ha in Manitoba CA (50° latitude) over a period of 21 years (1992–2012). Their model contained 17 variables, including precipitation, temperature, and snow depth during various periods. The significant precipitation variables in their model that explaining total flowering plant population size were previous mature, previous senescence, postsenescence, and emergence. All of these coefficients were positive, suggesting that more rainfall during these periods increased population size (as determined by number of flowering individuals). All three of these studies (Bleho et al., 2015); Morrison et al., 2015; Willson et al., 2006; ; , climate data were taken from a single weather station and observations began in the early 1990s and covered no more than 21 years. In contrast, the data used in this manuscript spanned 12.5° latitude (37.5°–50°), 34 weather stations, and 118 years (1894–2012).

Here, we modify those models used to explain WFPO population size to understand the contribution of precipitation to WFPO production, as characterized by plant height and reproductive effort recorded in herbarium specimens, which expands the temporal and spatial sampling of individuals. We explore several hypotheses. First, we hypothesize that our WFPO data may follow similar patterns found by previous models that describe plant population size as a function of precipitation during specific critical phenological periods (H1). Second, we compare the performance three precipitation summary models in describing WFPO height or reproductive effort (H2). Third, we hypothesize that plant height and reproductive effort will respond similarly to precipitation patterns (H3). Finally, we examine how latitude and collection year may contribute to WFPO height and reproductive effort (H4).

## 2 METHODS

### 2.1 Historical specimen dataset

To locate WFPO specimens, we searched the Great Plains Regional Herbarium Network and Consortium of Northern Great Plains Herbaria (ngpherbaria.org, accessed 7 November 2017) and also searched other herbaria databases throughout this region and directly contacted regional institutions for information. Although we endeavored to find all known specimens, some institutions were unresponsive.

We were able to acquire images from 21 herbaria, which included 242 herbarium sheets containing a total of 270 specimens. We did not have funding for travel or shipping, therefore, observing the actual specimens was not possible. Of these specimens, 130 lacked critical label information, such as date or location, or the specimen predated available climate data. Therefore, we were able to use 141 WFPO specimens in our analysis. Multiple specimens on the same sheet or from the same county in the same year were randomly assigned a replicate number. From each image, we determined plant height (cm) using ImageJ to measure both the 10 cm scale bar (photographed with the specimen) and the plant. We then used algebra to convert plant size to cm (Schneider, Rasband, & Eliceiri, 2012). Reproductive effort (flowers and buds) was estimated by two different individual researchers counting structures in the image. The few discrepancies were resolved through a recount. We also recorded the collection date and the county of collection^1^ and used Google Maps to determine the latitude of each county's centroid.

### 2.2 Climate data

Daily precipitation data (mm) for the locations of the historical WFPO locations were downloaded from Iowa State University's Environmental Mesonet (mesonet.agron.iastate.edu/climodat/, accessed 26 January 2018). For each location, we chose the closest weather station that was in operation at the time of plant collection. We then characterized precipitation in several ways. First, we used general phenological categories developed by Wolken (1995) and used, although not independently verified, by several researchers to model WFPO population size (Bleho et al., 2015, Morrison et al., 2015, and Willson et al., 2006). We modified these time periods to include environmental cues when possible, which reflects our observations from long‐term population monitoring across a broader, albeit still small, range of latitudes (Biederman et al., 2018). These categories include previous mature (PM), precipitation from 200 growing degree days (GDD) to 21 June in the previous year; previous senescence (PS), precipitation from 21 June to 31 August in the previous year; previous postsenescence (PP), precipitation from 1 September to 30 September in the previous year; dormancy/emergence (DE), precipitation from 1 October the previous year to 200 GDD the current year; and mature (CM), precipitation from 200 GDD to 21 June of current year.

Then, we calculated three summary measures to characterize precipitation over a longer time frame: accumulated, precipitation from 1 January to the specimen collection date; previous, precipitation from 1 January to 31 December in the year previous to collection; and Pre‐200, precipitation from 200 GDD in the previous year to 200 GDD in the current year.

### 2.3 Statistical analysis

All analyses were conducted in R (R Core Team, 2019), and data are available at Figshare (Biederman & Weldon, 2020). In 27 cases, there were multiple (up to 8) collections from the same population in the same year, either on the same sheet or sheets sent to multiple herbaria. To avoid pseudoreplication, we bootstrapped sampling so that only one individual per population and year combination was randomly selected for an individual analysis (*n* = 90). We then performed the analysis for 1,000 iterations using the R package “boot” (Canty & Ripley, 2020) and presented the average model coefficients and *R* ^2^.

We evaluated the relevance of the precipitation models developed by Willson et al. (2006), Morrison et al. (2015), and Bleho et al. (2015) in determining the characteristics of plant height and reproductive effort by using bootstrapped regression models (H1). We also used bootstrapped linear regression to directly compare the three precipitation summaries in their ability to affect WFPO height and reproductive effort (H2). The fit of these various models was compared using delta Akaike's information criterion (ΔAIC) (Burnham & Anderson, 2002). Pearson correlations were conducted to determine the relationship between WFPO height and reproductive effort (H3), and also among these variables and precipitation, latitude, and year of collection (H4).

## 3 RESULTS AND DISCUSSION

The specimens used in this analysis were collected between 1894 and 2012, from locations within Manitoba Province in Canada (6 specimens), and Iowa (47), Kansas (20), Minnesota (23), Missouri (3), Nebraska (14), and North Dakota (31) in the United States. The earliest collection day of the year was on 23 May and the latest was 21 August. WFPO plants in our dataset ranged between 20.4 and 84 cm in height and had between 1 and 21 reproductive structures (total count of flowers and/or buds present).

Hypothesis 1 Table 1 provides the results of our bootstrap analysis on herbarium specimens of Western Prairie Fringed Orchid (*Platanthera praeclara* Sheviak & Bowless) using models modified from Willson et al. (2006), Morrison et al. (2015), and Bleho et al. (2015). We find that this approach, while perhaps useful for describing population dynamics at a single site, does not translate to explaining plant height or reproductive effort across a range of sites and time.

**TABLE 1:** Results of the bootstrap analysis (*n* = 90) of herbarium specimens of western prairie fringed orchid (*Platanthera praeclara* Sheviak & Bowless) using models modified from Willson et al. (2006), Morrison et al. (2015), and Bleho et al. (2015)

	Willlson et al.		Morrison et al.		Bleho et al.	
	Height	Repro	Height	Repro	Height	Repro
*R* ^2^	.05	.00	.062	.022	.06	.01
Intercept	40.9	9.06	40.36	10.21	37.96	9.813
PM			0.037	0.006	0.014	−0.0065
PS	0.016	0.001			0.017	0.0026
PP					−0.008	−0.004
DE	0.036	0.002	0.017	−0.007	0.033	0.0058
CM						

Note

We examined the effect of precipitation during various phenological periods on plant height (cm) and reproductive effort (flower and bud number). Data include model *R* ^2^, intercept, and precipitation categories: previous mature (PM), precipitation from 200 growing degree days (GDD) to 21 June in the previous year; previous senescence (PS), precipitation from 21 June to 31 August in the previous year; previous postsenescence (PP), precipitation from 1 September to 30 September in the previous year; dormancy/emergence (DE), precipitation from 1 October the previous year to 200 GDD the current year; mature (CM), precipitation from 200 GDD to 21 June of current year.

**FIGURE 1:**
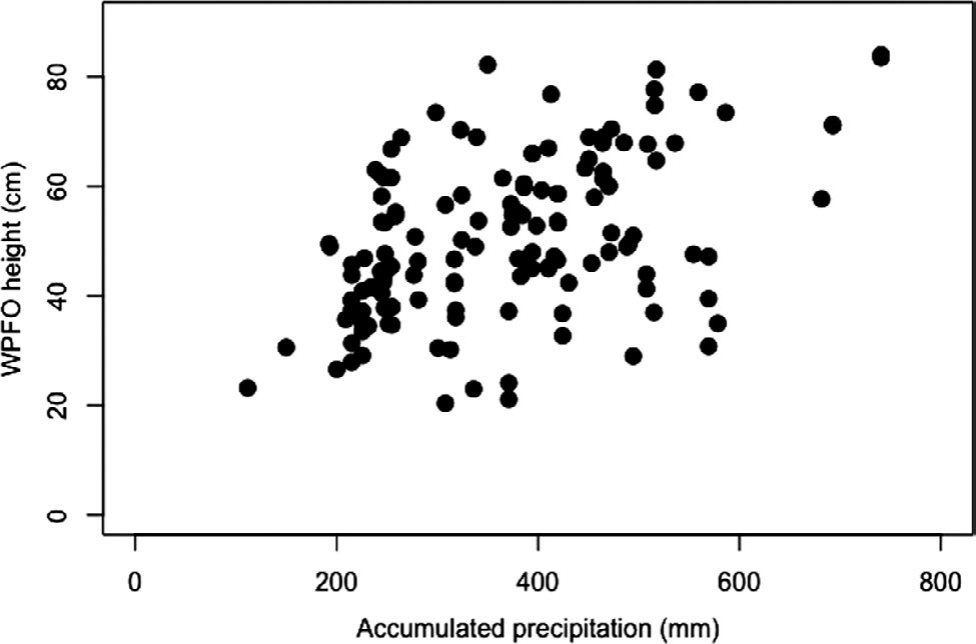
Western prairie fringed orchid (*Platanthera praeclara* Sheviak & Bowles; WFPO) height (cm) as a function of precipitation (mm) accumulated since 1 January of the collection year (*n* = 141)

Hypothesis 2 Delta AIC values of various precipitation summary models (Table 2) suggest that accumulated precipitation from 1 January to the specimen collection date was the best among the summarized precipitation variables for explaining plant height (Figure 1), although the mean *R* ^2^ of this model was low (.192). Although tuber development in the previous year is the carbohydrate resource for the following year's WFPO plants (Smith, 2012; Wolken, Hull Sieg, & Williams, 2001), recent conditions (as estimated by precipitation from the beginning of the calendar year) seem to be more important for plant growth than conditions during tuber development. In a recent model of plant growth and anthesis, precipitation needed to be adequate for WFPO flowering, as even short‐term droughts can halt plant growth and halt flower development (Biederman et al., 2018). We cannot differentiate among the candidate models for reproductive effort, as none were >2 AIC values from others (Burnham & Anderson, 2002).

**TABLE 2:** AIC, ΔAIC, and R^2^ values of the compared the bootstrapped linear models (*n* = 90) describing western prairie fringed orchid (*Platanthera praeclara* Sheviak & Bowles) plant height and reproductive effort with various precipitation parameters, including accumulated precipitation, which was occurred between 1 January to the specimen collection date; previous year precipitation, which accumulated from 1 January to 31 December in the year previous to plant collection; and Pre‐200, which described precipitation from 200 growing degree days (GDD) in the previous year to 200 GDD in the year of plant collection

	Height			Reproductive effort		
	AIC	ΔAIC	*R* ^2^	AIC	ΔAIC	*R* ^2^
Accumulated precipitation	726.2	0	.192	497.2	0.5	.000
Previous year precipitation	739.1	12.9	.067	496.7	0	.000
Pre‐200 precipitation	736.5	10.3	.094	497.1	0.4	.000

Hypothesis 3 Plant height and reproductive effort did not respond similarly to precipitation. In fact, reproductive effort did not respond to any of our tested precipitation parameters. Plant height and reproductive effort were weakly correlated (*r* = .248); taller plants generally had more reproductive structures than shorter plants. However, this correlation in plant height and flower number is smaller than the correlation between annual mean plant height and flower number (*r* = .49) found by Morrison et al. (2015).

Hypothesis 4 WFPO plants were generally shorter at higher latitudes (*r* = −.225). This may be the result of a shorter field season, which is constrained by both later attainment of 200 GDD and the static summer solstice (Biederman et al., 2018). Reduced accumulated precipitation at higher latitudes (*r* = −.464) may also have contributed to shorter plants.

WFPO plant height decreased somewhat with collection year (*r* = −.292). Although the central region of North America, which includes the range of WFPO, is expected to experience an overall increase in precipitation during early spring as the climate changes (Gutowski et al., 2008; U.S. Climate Change Science Program, 2008), our collection data suggest that accumulated precipitation between 1 January and collection date have generally decreased at collection sites since 1894 (*r* = −.451). Species that specialize in wetter habitats, such as WFPO, may be especially vulnerable to frequent drought conditions, which are expected to become more common in the future (Craine et al., 2011).

This analysis, although expansive over time (1894–2012) and latitude (37.5°–49.9°), has few observations (*n* = 90) and is therefore limited. Site and year specific conditions, such as drainage qualities and nutrient availability, as well as genetic characteristics of the population, also contribute to plant height and reproductive effort, and we were not able to assess these with our dataset. There may also be mutualistic interactions, such as those between WFPO and plant mycorrhiza infection, or lagged effects, such as with plant germination and early growth, that we know little about and cannot deduce from herbarium specimens (Rasmussen, Dixon, Jersakova, & Tesitelova, 2015). Furthermore, collectors may also have been biased in the choice of specimen (Lang, Willems, Scheepens, Burbano, & Bossdorf, 2018), picking the largest or most productive plants. Unfortunately, herbarium labels often lack this important information and revisiting sites is impossible as many of the populations have been extirpated.

Understanding how climate change will affect natural systems is a significant and growing challenge for scientists and natural resource managers (Hannah et al., 2002; Williams, Jackson, & Kutzbach, 2007). Limited financial and personnel resources constrain monitoring, yet understanding population characteristics is critical for conserving vulnerable species (Fay, Paillet, & Dixon, 2015; McLachlan, Hellmann, & Schwartz, 2007). Herbaria specimens, while imperfect, are collected over broad spatial and temporal scales and represent time capsules that allow us to infer species response to changes in climate based on their responses in the past (Jones & Daehler, 2018; Lang et al., 2018; Meineke, Davis, & Davies, 2018; Pearse, Davis, Inouye, Primack, & Davies, 2017). Expanding this analysis to other species, such as other members of the Orchidaceae, would further refine our understanding of plant response to change.

## CONFLICT OF INTEREST

We do not have any conflicts of interest.

## AUTHOR CONTRIBUTIONS

**Lori A. Biederman:** Conceptualization (lead); data curation (lead); formal analysis (lead); funding acquisition (lead); investigation (lead); methodology (lead); project administration (lead); resources (lead); software (lead); supervision (lead); validation (lead); writing – original draft (lead); writing – review and editing (equal). **Sydney M. Weldon:** Data curation (supporting); resources (supporting); writing – review and editing (supporting). **Derek S. Anderson:** Conceptualization (supporting); methodology (supporting); writing – review and editing (equal). **Mark J. Leoschke:** Conceptualization (supporting); methodology (supporting); writing – review and editing (supporting).

## Data Availability

Specimen and climate data are available at www.doi.org/10.6084/m9.figshare.12318590.

## ENDNOTE

^1^ Because the WFPO is rare and collectors constitute a threat, all population locations are identified to county and the latitudes used are the centroids of these counties (Biederman and Weldon 2020). Specific information may be obtained by contacting the authors.
